# Rituximab-Induced Serum Sickness in a Seven-Year-Old Child With Nephrotic Syndrome

**DOI:** 10.7759/cureus.88465

**Published:** 2025-07-21

**Authors:** Joaquin Mauvezin, Lorena Pardo, Andrea Cairus, Ilse Deutsch, Maite Inthamoussu, Gustavo Giachetto

**Affiliations:** 1 Pediatrics, Facultad de Medicina, Universidad de la República, Montevideo, URY; 2 Pediatric Nephrology, Facultad de Medicina, Universidad de la República, Montevideo, URY; 3 Pharmacology, Facultad de Medicina, Universidad de la República, Montevideo, URY

**Keywords:** child, drug side effects, nephrotic syndrome, pharmacovigilance, rituximab-induced serum sickness

## Abstract

The aim of this article is to report an unusual side effect of rituximab in a child. We describe a seven-year-old boy with steroid-dependent nephrotic syndrome who presented with fever and severe polyarthralgia after his first dose of rituximab. The onset of symptoms 13 days after rituximab administration, the self-limited evolution of symptoms, the exclusion of other causes, and the presence of similar reports led to the diagnosis of rituximab-induced serum sickness as a possible adverse reaction. The benefits of rituximab have been reported in steroid-dependent nephrotic syndrome with frequent relapses. However, most of the available evidence derives from studies in adults. The communication and analysis of pharmacovigilance data, both from spontaneous reporting and active surveillance, are important for defining the role of new drugs in treatment.

## Introduction

Nephrotic syndrome is a common kidney disease in pediatrics. While most children respond favorably to corticosteroids, some require alternative therapeutic approaches to minimize prolonged steroid exposure. Rituximab is a chimeric monoclonal IgG1 antibody that binds to CD20 molecules, leading to a reduction in peripheral B cells. It has been proposed as a treatment for steroid-sensitive nephrotic syndrome, as a second-line steroid-sparing agent in children with frequently relapsing nephrotic syndrome (FRNS) or steroid-dependent nephrotic syndrome (SDNS), and as a rescue therapy for multidrug-resistant steroid-resistant nephrotic syndrome (SRNS). The available evidence on its efficacy and safety is limited. Although it has been shown to reduce relapses and reduce the use of corticosteroids, there is no agreement on the dose to be used, the inter-dose interval, and the duration of use [1, 2].

Despite these uncertainties, rituximab has been approved for the treatment of nephrotic syndrome by some regulatory agencies. In this context, phase 4 clinical trials are essential to better define its therapeutic role and to assess its safety profile, including both known and unexpected adverse effects [2,3].

Common side effects described in their data sheets include all infusion-related reactions, which are usually immediate onset, including fever, chills, gastrointestinal symptoms, skin reactions, and hypotension, among others. Musculoskeletal symptoms such as myalgia and arthralgia are also frequently reported. Other less common adverse effects include thrombocytopenia, neutropenia, hypogammaglobulinemia, and cardiac or pulmonary disorders, with the most feared complications being fulminant myocarditis, lung fibrosis, and *Pneumocystis jiroveci *pneumonia [4,5]. The aim of this article is to report an unusual side effect of this medication in a child.

## Case presentation

A seven-year-old boy with steroid-dependent nephrotic syndrome presented with fever and severe polyarthralgia after his first dose of rituximab. The patient was diagnosed with steroid-dependent nephrotic syndrome at three years of age. Renal biopsy showed mild mesangial proliferation without segmental sclerosis or hyalinosis. Immunofluorescence was negative for IgA, IgG, IgM, C3, and C1q. Electron microscopy revealed hypertrophic podocytes with vacuolization and villous transformation, with foot process effacement affecting less than 40% of the capillary surface. No electron-dense deposits were observed.

He has received high doses of prednisone since being diagnosed and cyclosporine at 5 mg/kg/day, with frequent relapses, the last dose administered two months before he presented the symptoms in question. Given the persistence of frequent relapses despite high-dose prednisone and cyclosporine, along with signs of steroid-related toxicity, rituximab was initiated as a steroid-sparing strategy while in remission. At that time, proteinuria and albuminuria were within normal range, as was serum albumin. A single dose of rituximab 350 mg/m² was given without infusion reactions.

However, 13 days after the administration of rituximab, he presented to the emergency department with a short history of fever and severe bilateral hip arthralgia of sudden onset, which did not improve with common analgesics and impaired his walking. He didn't have a history of previous trauma, symptoms associated with connective tissue diseases, viral infections, or any other symptoms.

At physical examination, he was febrile, 38ºC, and presented with serious arthralgia on both hip rotations. No redness or swelling was present in the joints. Laboratory tests are shown in Table 1. 

**Table 1:** Laboratory findings NR: not reactive

Parameters	Results	Reference Range
Urine protein (spot)	0.05 g/L	0–0.15 g/L
Urine creatinine	52 mg/dL	39–259 mg/dL
Protein-to-creatinine ratio	0.10 g/g	0.00–0.20 g/g
Urinary albumin	<3 mg/L	0–20 mg/L
Serum albumin	4.30 g/dL	3.80–5.40 g/dL
Leukocytosis	14300/µL	4500-13500/µL
Neutrophils	9724/µL	1500-8500/µL
Platelets	355000/µL	150000-450000/µL
Hemoglobin	13,4 g/dL	12-14,5 g/dL
C reactive protein	9,2 mg/L	< 20 mg/L
Procalcitonin	<0,05 ng/mL	<0,05 ng/mL
Erythrocyte sedimentation rate	57 mm/h	< 15 mm/h
Prothrombin time	97%	76-104%
Activated partial thrombin time	32 seg	31-44 seg
Fibrinogen	349 mg/dL	200-390 mg/dL
Antinuclear antibodies (ANA)	NR	-
Anti-native DNA antibodies	NR	-
Rheumatoid factor	NR	-
C3	176 mg/dL	88-201 mg/dL
C4	38 mg/dL	15-45 mg/dL
Antistreptolysin O test	NR	-

The patient was admitted to the hospital and started on empiric antibiotics with clindamycin. The arthralgia spread to both knees, he could not walk, and required opioids for pain control during the first 24 hours.

**Figure 1:**
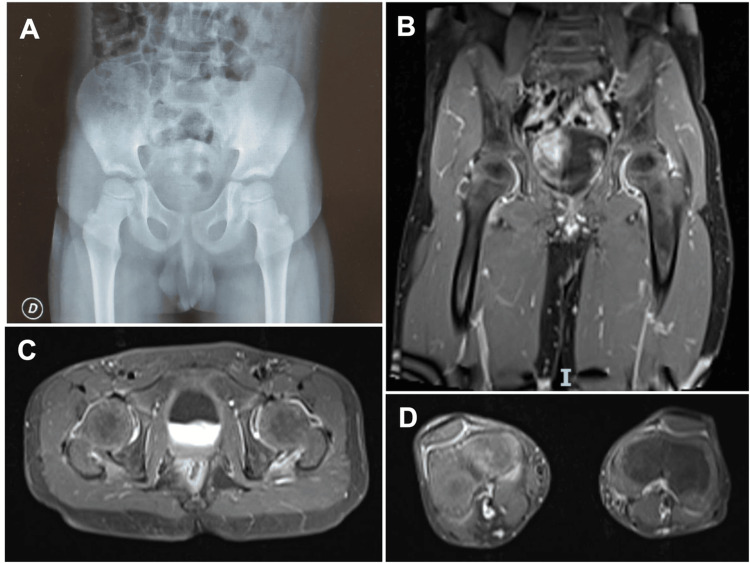
Imaging findings of the hip and knee joints A. Plain radiograph of the pelvis (anteroposterior view) showing no evident erosions or abnormalities in the hip joints. B, C. Coronal and axial MRI images of the pelvis demonstrating mild bilateral joint effusion and synovial enhancement in the hip joints, consistent with arthritis. D. Axial MRI of the knees showing synovial enhancement without evidence of joint effusion.

An MRI scan demonstrated articular effusion with synovial enhancement of both coxofemoral joints in relation to arthritis. It also showed synovial enhancement of both knee joints without evidence of effusion. No erosions or bone lesions were observed (Figure 1).

The fever remitted in 48 hours, and the pain resolved in three days. Blood, urine, and throat cultures were negative. Septic arthritis was ruled out, and antibiotics were suspended after three days. The beginning of an autoimmune disease was investigated, and tests for antinuclear antibodies (ANA), anti-native DNA antibody, and rheumatoid factor were not reactive. Erythrocyte sedimentation rate was 57 mm/h. His C3 level was 176 mg/dl, C4 level was 38 mg/dl, and the antistreptolysin O test was negative. Diagnostic tests for influenza A and B, respiratory syncytial virus (RSV), adenovirus, SARS-CoV-2, HIV, hepatitis B and C, cytomegalovirus (CMV), Epstein-Barr virus (EBV), and *Toxoplasma gondii *were all non-reactive.

Considering the onset of symptoms 13 days after the infusion of rituximab, with fever and self-limited polyarthritis, excluding other causes and using the “Naranjo algorithm” (proposed by Pan American Health Organization/World Health Organization (PAHO/WHO) in the document “Good Pharmacovigilance Practices for the Americas”) the diagnosis of rituximab-induced serum sickness was proposed as a possible side effect [4].

He was discharged after four days with cyclosporine and prednisone (1mg/k/day). Eight months later, during the follow-up visits, the patient exhibited significant improvement, reporting no pain and lacking any joint or systemic symptoms. Rituximab treatment was discontinued, and due to the patient's ongoing nephrotic relapses, tacrolimus was initiated as a replacement for cyclosporine, with no adverse effects reported to date.

## Discussion

Rituximab is being used more frequently, even in pediatric populations, for immune-mediated, hemato-oncologic diseases and steroid-dependent nephrotic syndrome. Despite the available data on its benefits and efficacy, which is acknowledged in the most recent pediatric nephrotic syndrome guidelines, the role of this medication in the treatment of nephrotic syndrome is not fully elucidated. The very common (1/10) and common (1/10 to 1/100) side effects described in their technical data sheets include all infusion-related reactions, fever, chills, nausea and vomiting, pruritus, angioedema, hypotension, headache, bronchospasm, urticaria, rash, myalgias, gastrointestinal disorders, fever, headache, skin rash, lymphopenia, neutropenia, myalgias and arthralgias. Other less common adverse symptoms include thrombocytopenia, hypogammaglobulinemia, and cardiac or pulmonary disorders [5].

Rituximab-induced serum sickness is a type III hypersensitivity reaction that occurs following the administration of foreign antigens, such as rituximab. This condition is characterized by the onset of fever, rash, arthralgia, adenopathies, and asthenia, typically occurring 7-14 days after drug administration. The majority of reported cases of rituximab-induced serum sickness occur in adults, with an average age of 38 years and a typical first dose of approximately 700 mg [6]. The most common clinical manifestations included articular symptoms (91.9%) and fever (86.5%). Patients with autoimmune disease often present with cutaneous manifestations, which were absent in this case [6,7].

Although the detection of human anti-chimeric antibodies contributes to the diagnosis, confirmation of “serum sickness due to rituximab” relies primarily on clinical symptoms and the exclusion of differential diagnoses [8].

In pediatric patients presenting with fever and joint effusion, the initial differential diagnosis typically includes infectious arthritis, particularly in an immunosuppressed patient. Given the high morbidity and mortality associated with bacterial infections in this type of host, initiating empirical antibiotic therapy targeting *Streptococcus aureus* is considered to be an adequate course of action in most of the schemes proposed for the approach of this entity. Although the primary diagnosis in this case was idiopathic nephrotic syndrome, secondary causes, including atypical manifestations of autoimmune diseases such as pediatric systemic lupus erythematosus, needed to be re-evaluated [9].

At the time of writing this communication, there were only five known reports of this adverse effect occurring in the pediatric population, also as a result of treatment for nephrotic syndrome [8,10-13]. In most cases, the approach included corticosteroids and antibiotics, and the symptoms regressed 48 hours after onset. Given the limited evidence, discussion between pediatricians and clinical pharmacologists was important to seek and analyze available information on the possible causal relationship between rituximab and this adverse event.

The risk-benefit analysis of continuing rituximab treatment, despite the risk of adverse effects, is critical, particularly given the severity of renal involvement. If the decision is made to continue this drug, close monitoring should be carried out in a hospital setting. Other modulators of the B lymphocyte response, such as belimumab, have been tried with lackluster results [14]. In the case of the patient reported, control of the relapses was achieved with tacrolimus, making it easier to discontinue treatment, as another safer and effective option is available.

## Conclusions

The communication and analysis of pharmacovigilance data, both from spontaneous reporting and active surveillance, are important to update recommendations and contribute to effectiveness, especially in off-label use cases, new drugs or new indications of use. 

This report describes the management of a rare rituximab-related adverse drug reaction in a child with nephrotic syndrome using the Naranjo algorithm and highlights the importance of pediatric pharmacovigilance in biologic therapies.
